# Interpretation of normal anatomic structures on chest radiography: Comparison of Fuji Computed Radiography (FCR) 5501D with FCR 5000 and screen‐film system

**DOI:** 10.1120/jacmp.v4i1.2547

**Published:** 2003-01-01

**Authors:** Kazuaki Nakashima, Kazuto Ashizawa, Makoto Ochi, Rashid Hashmi, Kuniaki Hayashi, Shinichi Gotoh, Sumihisa Honda, Akito Igarashi, Takao Komaki

**Affiliations:** ^1^ Department of Radiology Nagasaki University School of Medicine 1‐7‐1 Sakamoto Nagasaki 852‐8501 Japan; ^2^ Division of Clinical Radiology Nagasaki University Hospital 1‐7‐1 Sakamoto Nagasaki 852‐8501 Japan; ^3^ Department of Radiation Epidemiology Nagasaki University School of Medicine 1‐12‐4 Sakamoto Nagasaki 852‐8523 Japan; ^4^ Fuji Medical Systems Tokyo Japan

**Keywords:** digital radiography, chest radiography, Fuji Computed Radiography, imaging plate

## Abstract

The purpose of this study was to investigate the usefulness of Fuji Computed Radiography (FCR) 5501D by comparing it with FCR 5000 and a screen‐film system (S/F). Posteroanterior chest radiographs often patients with no abnormality on chest CT scans were obtained with FCR 5501D, FCR 5000, and S/F. Six observers (three radiologists and three radio‐technologists) evaluated the visibility of nine normal anatomic structures (including lungs, soft tissue, and bones) and overall visibility on each image. Observers scored using a five‐point scale on each structure. FCR 5000 showed a significantly higher score in soft tissue and bone structures, and overall visibility compared with S/F, but, there was no significant difference between them in the visibility of all four normal lung structures. Compared with S/F, the score for FCR 5501D was higher in eight of the nine normal structures, including three of the four lung structures (unobscured lung, retrocardiac lung, and subdiaphragmatic lung), and overall visibility. Compared with FCR 5000, the score for FCR 5501D was higher in three normal structures, including two of the four lung structures (unobscured lung and subdiaphragmatic lung), and overall visibility. FCR 5501D was the best among the three techniques to visualize normal anatomic structures, particularly the obscured and unobscured lung. © *2003 American College of Medical Physics.*

PACS number(s): 87.57.–s, 87.62.+n

## INTRODUCTION

Fuji Computed Radiography (FCR) is a digital radiographic system generally used for chest imaging. Similar to other digital radiographic systems, FCR has eminent characteristics such as wide dynamic range and flexible postprocessing.^1^–^5^

**Figure 1:**
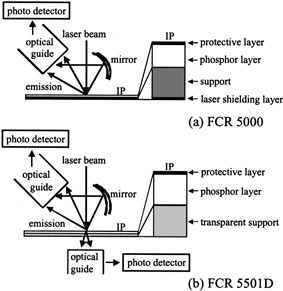
Diagrams of Fuji Computed Radiography (FCR). (a) FCR 5000 derives the laser information from one side of the imaging plate (IP). (b) FCR 5501D adopts a thickened phosphor layer and transparent support on the IP, and reads both sides of the IP.

Newly developed FCR 5501D adopts a thickened storage phosphor layer and transparent support, and scans both sides of a phosphor screen, imaging plate (IP), with a finely focused laser beam6 (Fig. 1). The phosphor layer of FCR 5501D is about 30% thicker than that of the conventional single‐side light collection IP. The amount of stored x‐ray energy on the IP increases with increasing thickness of the photostimulable phosphor layer. However, with the conventional single‐side IP scanning, the emission deep in the phosphor layer is difficult to detect on the front side of the IP. Excess thickness of the phosphor layer is thus not efficacious for optimal use of x‐ray. FCR 5501D improves this issue by detecting laser‐stimulated luminescence in the deep part of the phosphor layer effectively from back of the IP through the transparent support. The data detected by each side (front and back) of the photo‐detector are added together using appropriate additive ratios. The combined final image data of this dual light collection system supplies more information than single‐side light collection IP. Because the emissions detected from the back side primarily contain data of low spatial frequency domain, FCR 5501D is expected to improve the image quality particularly in the low spatial frequency areas. Using this technique, the signal‐to‐noise ratio of the image is anticipated to improve compared with conventional FCR, such as FCR 5000, which derives information from the single side of the IP.

In this study, we investigate the usefulness of FCR 5501D in the visualization of normal anatomic structures, comparing with FCR 5000 and a dual‐emulsion screen‐film system (S/F).

## MATERIALS AND METHODS

### A. Study group

**Figure 2:**
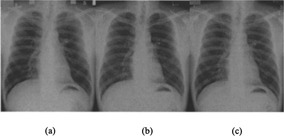
Representative set of chest radiographs of a 42‐year‐old man. (a) Screen‐film, (b) FCR 5000, and (c) FCR 5501D.

The study group included ten patients (four women and six men; age range, 36–74 years; mean, 56 years) who underwent chest CT scans showing no abnormality. The CT studies were carried out for varied clinical indications: respiratory symptoms (n=4), suspected esophageal cancer (n=2), malignant lymphoma, Sjoegren's syndrome, chest pain, and asymmetric brachial blood pressure. In each patient, FCR 5501D, FCR 5000, and S/F images were obtained on the same day (Fig. 2). The interval between chest radiography and CT was one week or less. Informed consent was obtained from all patients.

### B. Image acquisition

Posteroanterior chest radiographs of FCR 5501D, FCR 5000, and S/F were acquired with the same exposure factors: tube voltage of 105 kVp, 8.0 mAs, 0.3 mm focus, and 200 cm film‐focus distance. FCR 5501D and FCR 5000 images were obtained using a commercial system (Fuji Medical Systems, Tokyo, Japan). The images were printed on a 35×43 cm film (CR 780‐H, Fuji Medical Systems, Tokyo, Japan) by a laser printer (CR‐LP D, Fuji Medical Systems, Tokyo, Japan). Each image has a matrix of 10 pixels per millimeter.

S/F radiographs were obtained using an automatic chest system (Philips Medical Systems, Hamburg, Germany) and an orthochromatic screen with dual‐emulsion film (Insight, IS‐952, Eastman‐Kodak, Rochester, USA).

### C. Reading methods

Ten sets of the FCR 5501D, FCR 5000, and S/F images were compared as the following combinations: S/F versus FCR 5000, S/F versus FCR 5501D, and FCR 5000 versus FCR 5501D.

Six observers (three radiologists and three radio‐technologists) evaluated the visibility of nine normal anatomic structures (airway, unobscured lung, retrocardiac lung, subdiaphragmatic lung, mediastinum, chest wall, abdomen, spine, rib, and shoulder girdle) and overall visibility (Table I). During interpretation, the paired images were placed on a view box and areas surrounding the films were covered by black paper to shield excess light.

Observers scored with a five‐point scale on each structure using a grading system described by Woodard *et al.* ^7^ (Table I). The images were given to the observers without any information, being simply named image *A* or *B.* The five‐point scale was defined as the following: 1=image A much better, 2=image A slightly better, 3=no difference between *A* and *B*, 4=image B slightly better, 5=image B much better. Intermediate scores at 0.5 were allowed; thus, there were nine possible scores. The scores of the six observers were averaged for each structure and for each patient, and the mean scores were calculated. Statistical significance was evaluated with a paired *t* test.

**Table I:** Score sheet for the evaluation of nine normal anatomic structures and overall visibility. 1=image A much better, 2=image A slightly better, 3=no difference, 4=image B slightly better, 5=image B much better.

Region	Image *A* vs Image *B*
1. Lung
Airway	1‐1.5‐2‐2.5‐3‐3.5‐4‐4.5‐5
Unobscured lung	1‐1.5‐2‐2.5‐3‐3.5‐4‐4.5‐5
Retrocardiac lung	1‐1.5‐2‐2.5‐3‐3.5‐4‐4.5‐5
Subdiaphragmatic lung	1‐1.5‐2‐2.5‐3‐3.5‐4‐4.5‐5
2. Soft tissue
Mediastinum	1‐1.5‐2‐2.5‐3‐3.5‐4‐4.5‐5
Chest wall	1‐1.5‐2‐2.5‐3‐3.5‐4‐4.5‐5
Abdomen	1‐1.5‐2‐2.5‐3‐3.5‐4‐4.5‐5
3. Bone
Spine	1‐1.5‐2‐2.5‐3‐3.5‐4‐4.5‐5
Ribs and shoulder girdle	1‐1.5‐2‐2.5‐3‐3.5‐4‐4.5‐5
4. Overall visibility	1‐1.5‐2‐2.5‐3‐3.5‐4‐4.5‐5

Results are summarized in Table II. FCR 5000 showed significantly higher score in soft tissue and bone structures, and overall visibility compared with S/F. However, there was no significant difference in the visibility of all 4 normal lung structures between FCR 5000 and S/F.

**Table II:** Comparative evaluation of visibility of normal anatomic structures among S/F, FCR 5000, and FCR 5501D. *=Statistically significant (p<0.05), NS=not significant.

	S/F vs FCR5000		S/F vs FCR5501D		FCR5000 vs FCR5501D	
Region	Mean score	p value	Mean score	p value	Mean score	*p* value
1. Lung						
Airway	2.94	NS	2.99	NS	3.03	NS
Unobscured lung	3.19	NS	3.49^*^	<0.001	3.21^*^	<0.001
Retrocardiac lung	3.00	NS	3.21^*^	0.016	3.03	NS
Subdiaphragmatic lung	3.00	NS	3.27^*^	<0.001	3.18^*^	<0.001
2. Soft tissue						
Mediastinum	3.17^*^	0.034	3.33^*^	<0.001	3.09	NS
Chest wall	3.13	NS	3.22^*^	<0.001	3.06	NS
Abdomen	3.27^*^	<0.001	3.24^*^	<0.001	3.04	NS
3. Bone						
Spine	3.83^*^	<0.001	3.79^*^	<0.001	3.13^*^	0.010
Rib and shoulder girdle	3.32^*^	<0.001	3.38^*^	<0.001	3.04	NS
4. Overall visibility	3.23^*^	0.014	3.48^*^	<0.001	3.23^*^	<0.001

Comparing FCR 5501D with S/F, the score for FCR 5501D was higher in eight of the nine normal structures, including three of the four lung structures (unobscured lung, retrocardiac lung, and subdiaphragmatic lung), and overall visibility.

Comparing FCR 5501D with FCR 5000, the score for FCR 5501D was higher in three normal structures, including two of the four lung structures (unobscured lung and subdiaphragmatic lung), and overall visibility.

## DISCUSSION

Because of large variations of x‐ray attenuation in the chest, which include structures such as lung, soft tissue, and bones, the state of the art digital chest radiography system is now equipped with wide dynamic range detectors. Many kinds of digital systems which use the luminescent phosphor technique, selenium‐based technique, scanning multiple beam equalization, etc. have been developed for improving the visibility of different structures in the chest. Numerous studies have shown efficacy of these system.^1^–^5^,^7^–^13^ FCR system adopts storage phosphor technique and is widely used for chest radiography. Its main characteristics are a slightly lower detective quantum efficiency (DQE) than conventional film, moderate spatial resolution, and wide latitude.^1^,^2^,^5^,^9^ Conventional FCR including FCR 5000, adopts single‐side IP scanning. By contrast, FCR 5501D features a transparent IP support, a thicker photostimulable phosphor layer, and a dual light collection image reading method. According to vendor specification, DQE of FCR 5501D is approximately 30–40% more compared to the single‐side reading method.^14^

Dual‐emulsion S/F is characterized by an excellent modulation transfer function (MTF), moderately good DQE, and limited latitude.^8^,^14^,^15^ FCR is superior to conventional radiography, especially in visualization of the mediastinum and the areas behind heart and diaphragm.^1^,^4^,^9^,^10^,^13^ In our study, FCR 5501D was superior to S/F in visualization of almost all chest anatomical structures. FCR 5501D was also superior to FCR 5000, in visualization of obscured and unobscured lungs.

For visualization of these areas, there was no significant difference between FCR 5000 and S/F. These results indicated that FCR 5501D improved the visibility of both of obscured and unobscured lungs. Although appropriate tube voltage for S/F may be controversial, we used relatively lower tube voltage (105 kVp) as a higher peak kilovoltage setting is known to result in lower contrast and loss of clarity of lung parenchyma.^8^,^14^,^16^ FCR 5501D was superior in the visualization of unobscured lung detail compared with S/F despite the advantage of lower tube voltage of S/F. Improvement of FCR 5501D in unobscured lung visibility matches with vendor specification of improvement of low spatial frequency area.

Even after the removal of alphanumeric and other information on the images, the FCR image could be differentiated from S/F. This could have resulted in observer bias and is one of the limitations of our study. Such bias is inevitable and may have occurred in the previous studies which studied visibility of normal anatomic features of the chest.^7^,^8^ However, it is difficult to differentiate FCR 5501D from FCR 5000 at a glance, hence the likelihood of observer bias is negligible. Another limitation of our study is the limited number of normal subjects. Factors such as degree of inspiration could cause a subtle difference in films of the same patient and could effect an observer's reading. Inclusion of a larger number of subjects could help minimize such influences.

In some phantom and clinical studies which assessed the visibility of pathologies, FCR showed equivalent or superior diagnostic capability compared with conventional radiography in nodular opacities; however, FCR was not superior to S/F in interstitial opacities.^1^–^4^,^5^ In another study, FCR was inferior to the S/F system in detectability of nodular shadows.^9^ FCR 5501D can possibly improve the visibility of such pathologies. In this study, we assessed the clinical usefulness of FCR 5501D only in normal subjects, and further investigation in pathological subjects should be performed.

## CONCLUSION

Among the three techniques, FCR 5501D is the best to visualize normal anatomic structures. In particular, it improves the visibility of both the obscured and unobscured lung structures.
